# Taxonomy based performance metrics for evaluating taxonomic assignment methods

**DOI:** 10.1186/s12859-019-2896-0

**Published:** 2019-06-11

**Authors:** Chung-Yen Chen, Sen-Lin Tang, Seng-Cho T. Chou

**Affiliations:** 1 Department of Information Management, National Taiwan University, Taipei, 106 Taiwan; 2 Biodiversity Research Center, Academia Sinica, Taipei, 115 Taiwan

**Keywords:** Metagenomics, Classification, Performance evaluation, Data analysis

## Abstract

**Background:**

Metagenomics experiments often make inferences about microbial communities by sequencing 16S and 18S rRNA, and taxonomic assignment is a fundamental step in such studies. This paper addresses the weaknesses in two types of metrics commonly used by previous studies for measuring the performance of existing taxonomic assignment methods: Sequence count based metrics and Binary error measurement. These metrics made performance evaluation results biased, less informative and mutually incomparable.

**Results:**

We investigated weaknesses in two types of metrics and proposed new performance metrics including Average Taxonomy Distance (ATD) and ATD_by_Taxa, together with the visualized ATD plot.

**Conclusions:**

By comparing the evaluation results from four popular taxonomic assignment methods across three test data sets, we found the new metrics more robust, informative and comparable.

## Background

### Taxonomic assignment using 16S and 18S rRNA gene classification

A fundamental step in microbiota studies is taxonomic assignment, in which each sequence or “read” in the study sample is assigned a taxonomic label [1]. The most common method for taxonomic assignment is to sequence the 16S and 18S rRNA genes as biomarkers, and there are several methods for doing this, including the RDP Naive Bayesian Classifier [2] (hereafter RDPNBC), K-Nearest Neighbor, SINTAX [3], TACOA [4], Taxator-tk [5], Kraken [6] and 16S Classifier [7]. Method performances are (cross-) validated on popular databases and have been characterized as having different strengths. Vinje et al. [8] compared performances for several k-mer based taxonomic assignment methods and found that the k-mer based methods that they used approach an error plateau.

### Challenges in performance evaluation

Taxonomic assignment methods are more difficult to evaluate than previously thought for several reasons:

1. Taxonomy Choice: Classification results using different taxonomic databases cannot be directly compared [9]. Since different sets of reference sequences and nomenclatures (e.g., Bergey’s, NCBI) are used, they might give the same taxonomic assignment for different query sequences or vice versa. Besides, taxonomic names are changed (or updated) as new microorganisms are identified, which makes the results even less consistent.
2. Testing Data: Data from communities differ from context to context (human gut, soil…etc.), and there are currently no standard testing data for each context. Previous studies derived their evaluation results by performing cross-validation on existing 16S and 18S rRNA databases such as RDP [10], Greengenes [11] and SILVA [12]. The Critical Assessment of Metagenome Interpretation (CAMI) [13] open-access platform also provides specially-generated data sets for benchmarking.
3. Reference database coverage: microbial marker genes such as 16S and 18S rRNA correspond to only a small fraction of species’ taxonomic names and known sequences [14]. Taxonomic assignment methods cannot learn the patterns from unseen taxa, regardless of their performance.
4. Performance Metrics: After cross-validating on databases, one may summarize the test results with some performance metrics such as accuracy, precision or recall. The different choices of metrics also reflect different viewpoints for the task and would reflect heavily on how researchers interpret the performance evaluation results. We believe that good taxonomic assignment performance metrics could help make inferences on the absolute performance given the known reference sequences and compare the performances among different methods. It is also worth mentioning that performance metrics are separate from the first three challenges because they have a stronger connection to referencing data sets. Performance metrics will always be the final direct performance reference for taxonomic assignment methods.

### Two weaknesses of performance metrics in previous studies

Previous studies showed that most taxonomic assignment algorithms could achieve around 90% accuracy when choosing genus as its classification target rank. High accuracy, however, does not necessarily imply high performance. Here, we illustrate two weaknesses of the performance metrics used by previous studies: Sequence Count Based Metrics and Binary Error Measurement.

#### Sequence count based metrics

##### Description

The performance metrics commonly used in previous studies, such as accuracy, precision or recall, are generally in the form of a fraction based on the count of predictions, such as:

$$ \mathrm{Accuracy}=\frac{N_{correct}}{N_{total}} $$

**Fig. 1:**
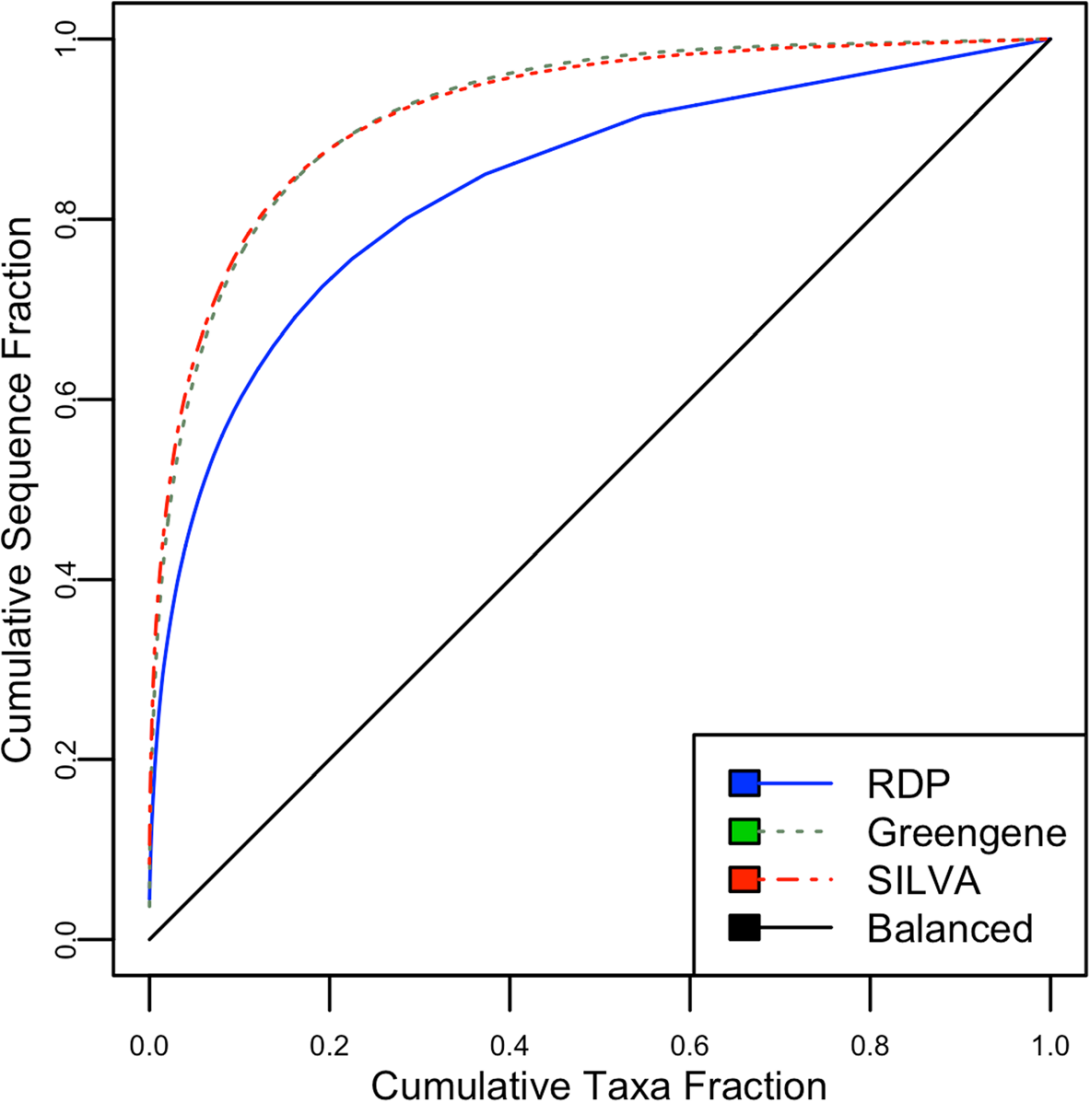
Cumulative sequence fractions for the common 16S and 18S imbalanced databases. (A balanced data set would assume a 45-degree line)

However, high accuracy does not necessarily represent good recognition capabilities in the classification task [15], especially when evaluating performance using imbalanced data sets. Data sets are imbalanced when some classes are highly underrepresented compared to others [16]. The performance evaluation of a classification model for multi-class imbalanced data sets in terms of simple “accuracy rate” may provide misleading results [17]. Unfortunately, common 16S and 18S databases are highly imbalanced. See Fig. 1 for cumulative sequence fractions in taxonomies (in their lowest rank) for the common databases. When evaluating performance on such imbalanced data sets, a result of 80% accuracy seems sufficient for a classification method at first glance, but it might accurately recognize only one-third of the taxa – an accuracy paradox.

##### Pitfalls

1. Biased Performance Evaluation

With sequence count based metrics, one may assume that the taxa distributions in databases are similar to those in samples, but this is usually not true in practical microbiota research. With regard to imbalanced data sets, the sequence count based metrics are just measuring how well a method performs based on a few specific taxa with high sequence frequency in a database, not its ability to recognize every taxon.

As a consequence, the performance evaluation results are optimistically biased toward the performance on high frequency taxa. The sequence count based metrics also favor methods that are good at recognizing patterns of high frequency taxa in databases.

(2) Incomparable Evaluation Results

To address the problem of frequent taxa, some previous studies resorted to “pruning” (undersampling) large taxa in databases to make the sequence counts for each taxon even [7, 8]. This strategy alleviates the imbalances in databases while trading off the sequence diversities for the pruned taxa, making the database coverage even poorer. Nevertheless, different undersampling methods in different studies make experiment results between studies mutually incomparable. Also, the vagueness in descriptions on how this pruning was done made the experiments less repeatable and reproducible.

##### Solution

Replacing the “taxa distributions in databases and samples are similar” assumption, we normalize taxa distributions by weighting each taxon equally in performance metrics to reflect a classification method’s recognition capabilities. We aim to give equal treatment to the prediction results of each taxon while avoiding resampling, which tends to make questionable adjustments to the original databases. In contrast to sequence count based metrics, this approach can be considered as “taxon count based metrics”. This concept has also appeared in some recent work [4, 5, 13].

#### Binary error measurement

##### Description

Literatures show that it is very common to measure prediction error in a binary form using:

$$ \mathrm{Per}-\mathrm{prediction}\ \mathrm{error}=\left\{\begin{array}{c}1, if\ incorrect\\ {}0, if\ corr\mathrm{e} ct\kern1em \end{array}\right. $$

without considering the similarities between taxa; the binary error measurement only takes equality into account. However, is mistaking Archaea as Bacteria (different domains) equally as wrong as mistaking Colwellia as Thalassomonas (different genera)?

**Fig. 2:**
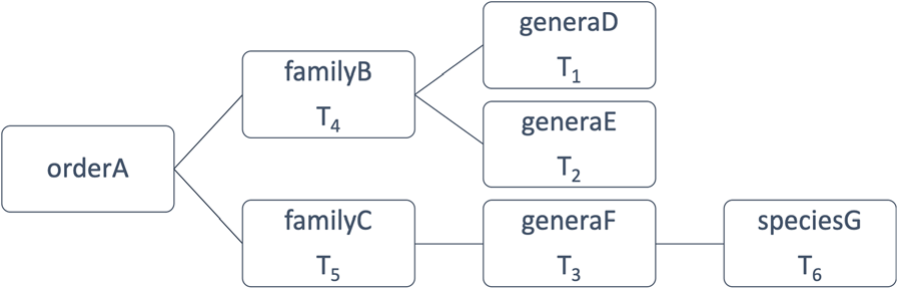
An example of a taxonomic tree. (For simplicity, only four ranks shown)

Consider the example illustrated in Fig. 2. Suppose there is a sequence with an actual taxonomic label T_1_“orderA;familyB;generaD”; T_2_ “orderA;familyB;generaE” and T_3_“orderA;familyC;generaF” are two predictions. Which prediction is better? Prediction T_2_ is “closer” to the actual taxon than Prediction T_3_ in number of different clade ranks. The binary error measurement would, however, treat both predictions equally.

##### Pitfalls

1. Loss of Information

The assumption behind the binary error measurement is that all taxa (taxonomic labels) are equally different from one another. But such an assumption does not bode well with the very nature of tree-based taxonomy where we view taxonomic assignment as a hierarchical classification (HC) problem. A hierarchical performance measure should use class hierarchy to properly evaluate HC algorithms [18].

Most previous studies made independent binary evaluations at each rank, in which performances were measured separately with different taxonomic ranks as classification targets [2, 4, 5, 13]. This design does not fully deploy the concept of HC, leading to loss of information as explained below.

When setting a high rank as the classification target, the evaluation result loses the information about whether a method is capable of differentiating the taxa in lower ranks. However, when setting a low rank as the classification target, we face the issue of singletons. Since singletons cannot be correctly classified, some previous studies discarded these predictions in statistics (making results overly optimistic).

Nevertheless, no taxon is completely novel in a taxonomic tree. Therefore, a method could still make generalized predictions on singletons. Discarding or ignoring them actually leads to the data diversities shrinking further and losing information on the performance on these variations.

Consequently, the performance evaluation results lose the information for prediction errors. The binary error measurement also does not favor “stable” methods (i.e., making fewer correct but overall fewer severely incorrect predictions); one example is the evaluation results presented in Vinje et al. (Fig. 5 in the paper), in which one cannot tell whether all compared methods made the same degree of mistakes in their common error predictions.

(2) Incomparable Evaluation Results

Previous studies viewed singletons as unavoidable (equal-degree) errors and used various treatments on these sequences. Therefore, using binary error not only caused loss of information, but raised the redundant issue for treatments on singletons, making the evaluation results incomparable.

We found that inconsistencies also existed within the same studies. For example, Fig. 1 in Wang et al.’s study [2] suggested one would lose merely 3% accuracy when changing the target rank from family to genus, but the evaluation results were actually based on different data sets (i.e., with different set of records recognized as singletons).

##### Solution

We change the “All taxa are mutually equally different” assumption by considering the dissimilarity between taxa to be proportional to their rank difference. We therefore define Taxonomy Distance as a way to measure the dissimilarity between any two taxonomic labels:

$$ TD=\frac{Number\ of\ ranks\ in\ difference}{\mathrm{N} umber\ of\ unique\ ranks\ in\  two\  taxa} $$

Consider again the taxonomic tree in Fig. 2. The calculation examples are shown in Table 1.

**Table 1:** An example TD calculation. (T_1.._T_6_ are the 6 taxonomic labels shown in Fig. 2)

*TD*	T_1_	T_2_	T_3_	T_4_	T_5_	T_6_
T_1_	0	1/3	2/3	1/3	2/3	3/4
T_2_		0	2/3	1/3	2/3	3/4
T_3_			0	2/3	1/3	1/4
T_4_				0	1/2	3/4
T_5_					0	2/4
T_6_						0

## Methods

We use the two solutions to propose a new set of performance metrics, together with a visualized plot, and reevaluate the performance of a few taxonomic assignment methods on three databases.

### Taxonomy based performance metrics

#### Per-prediction error: Taxonomy Distance

For a given query sequence, a taxonomic assignment method gives a taxonomic label as a prediction. The Taxonomy Distance in a prediction is *TD* as defined above.

#### Per-taxon error: Average Taxonomy Distance

The prediction error for a taxon T, called its “Average Taxonomy Distance” (ATD), is defined as

$$ ATD=\frac{\sum \limits_{i=1}^N TD\left({s}_i,P\left({s}_i\right)\right)}{N} $$

Where

$$ N= Number\ of\ sequences\ truly\kern0.5em in\ taxon\ T $$

$$ \left\{{s}_1,{s}_2,{s}_3\dots {s}_N\right\}= Sequences\ truly\ in\ taxon\ T $$

$$ \kern1.5em P(s)= The\ predicted\ taxon\ label\ for\ sequence\ s $$

#### Overall performance: ATD_by_Taxa

We used ATD_by_Taxa to measure the overall performance for a taxonomic assignment method, which is the simple mean of the ATDs for all the taxa

$$ \mathrm{ATD}\_\mathrm{by}\_\mathrm{Taxa}=\frac{\sum_{i=1}^M ATD\left({T}_i\right)}{M} $$

Where

$$ M= Total\ number\ of\ taxa $$

#### Error Rate (by taxa) and ATD (by seq)

We also derived two metrics to compare the effects of our two solutions: Err_by_taxa and ATD_by_seq, which use only one of the two solutions. Error rate (by taxa) is a taxon count based metric that uses binary error for each prediction, in which error rates are calculated for each taxon and then averaged. ATD (by seq) is a metric using Taxonomy Distance, but with no reference to taxon count; it is simply the mean of TDs among the predictions.

#### Visualizing through an ATD plot

**Fig. 3:**
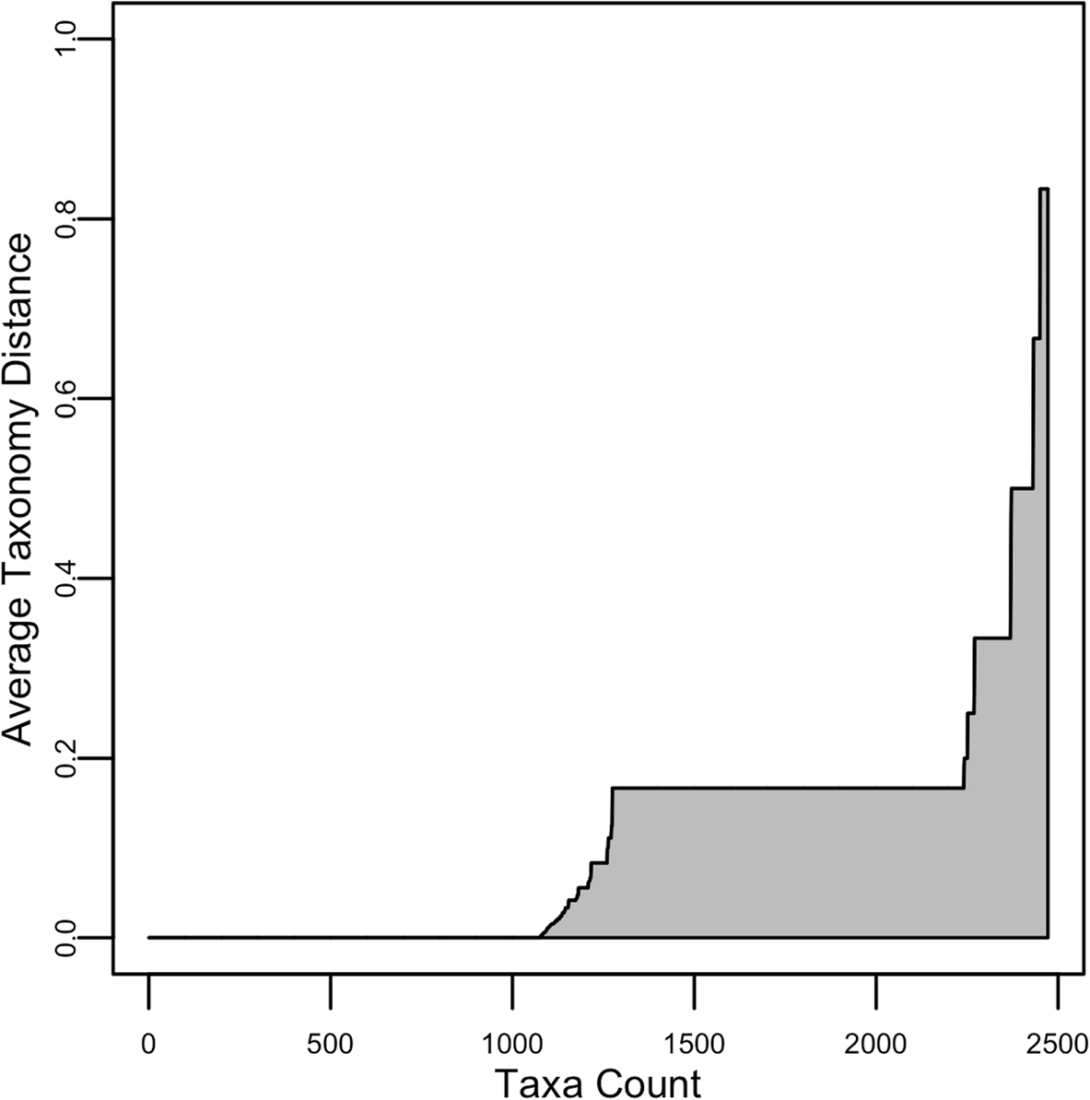
Example of ATD plot. This example plot shows that the method correctly classified around 1/2 of the taxa in the RDP database and around 1/3 of the taxa with 0.16 TD (1-rank error)

A taxonomic assignment can be visualized through a graph, or ATD plot, which is the plot of taxa ATDs sorted in ascending order. This plot shows the degrees of differences between predicted and actual taxa. See Fig. 3 as an example.

### Data and taxonomic assignment methods

#### 10-fold cross-validation and macro average

We used stratified 10-fold cross-validation for this study to reduce the outcome variance and bias across the folds [19]. In keeping with the uniform taxa distribution assumption, we performed macro-average [20] rather than micro-average when summarizing ATDs for each taxon. That is, rather than calculate performance metrics for each data fold and average them, we first aggregated all the TDs from the data folds, then calculated ATDs for each taxon.

#### Data

We chose RDP, Greengenes and SILVA as our testing data to evaluate 16S and 18S rRNA taxonomic assignment methods. They were all downloaded from Mothur.org [21]. Mothur had added some mitochondrial sequences from eukaryotes and removed subranks in the RDP labels. They also removed some non-16S, chimeric or low-quality sequences in SILVA. A detailed description of these data sets can be found on Mothur’s wiki page. Table 2 summarizes some characteristics of these databases.

**Table 2:** Summary for the rRNA gene databases used for this study

Database	Version	Sequence Type	Sequences	Taxa	Singletons
RDP	V16	16S	13,212	2472	1119
Greengenes	Aug2013	16S	203,452	5405	2078
SILVA	V128	16S & 18S	190,061	2078	1920

This study used the full length 16S and 18S rRNA gene sequences throughout the training and testing processes, and no singletons or other sequences were discarded from the databases so as to keep results comparable and maintain sequence variation.

#### Taxonomic assignment methods

Four taxonomic assignment methods with settings shown in Table 3 were chosen for this study. K-Nearest Neighbor (hereafter KNN), Nearest Neighbor (hereafter 1NN) and RDPNBC were implemented by Mothur. SINTAX was from USEARCH. For better evaluation results, we set the cutoff for RDPNBC and SINTAX to 0.

**Table 3:** Settings for the chosen taxonomic assignment methods

Method	Word length	Other parameters	Implemented by
KNN	8		Mothur v.1.39.5
1NN	8	numwanted = 1	Mothur v.1.39.5
SINTAX	8	cutoff = 0	USEARCH v9.2
RDPNBC	8	cutoff = 0	Mothur v.1.39.5

## Results

### The effects of taxon count based metrics and taxonomy distance

**Fig. 4:**
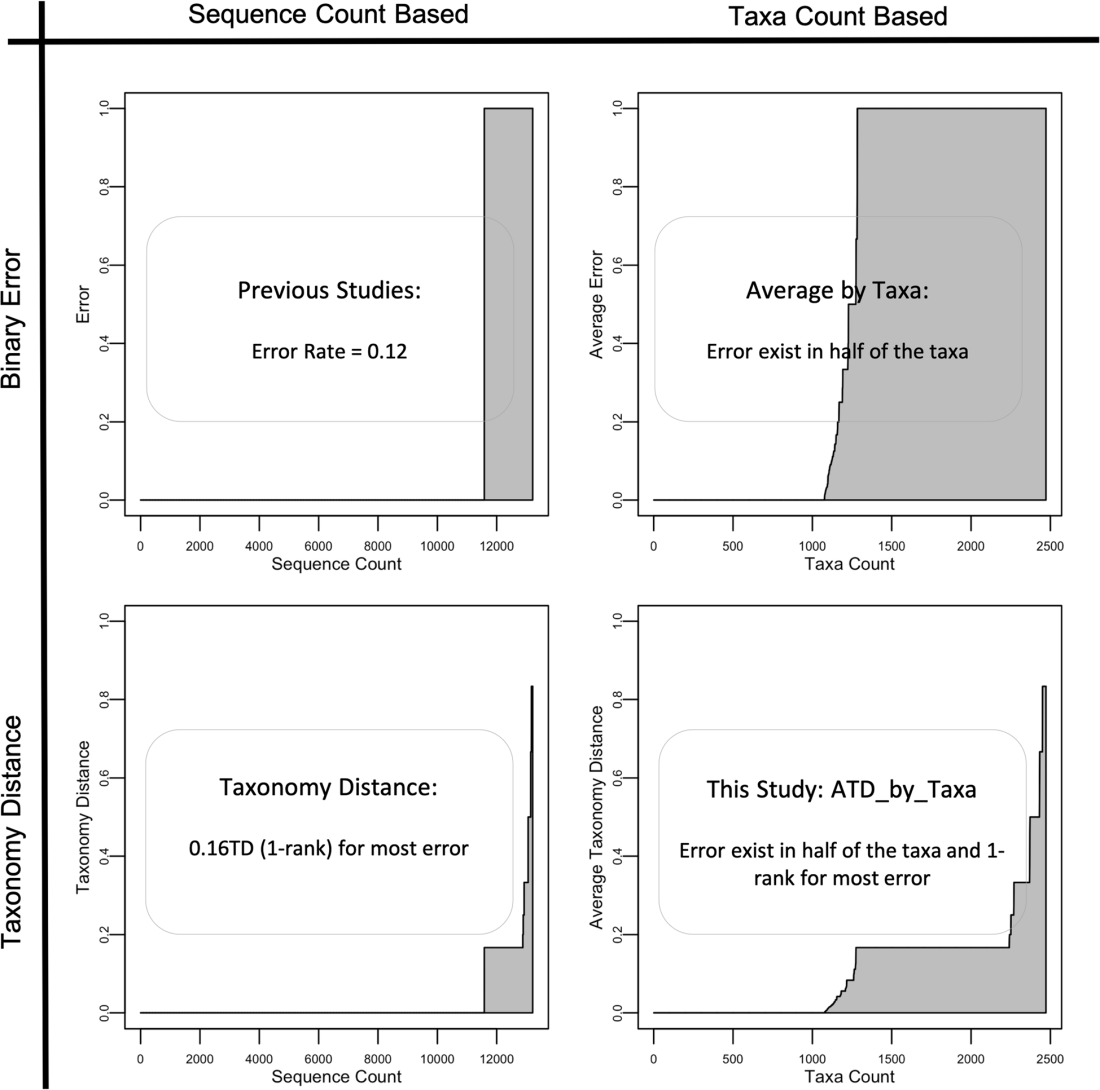
Evaluation results from testing RDPNBC on the RDP database

In order to know the effects from taxon count based metrics and Taxonomy Distance, we compared results from 2 × 2 cases. Figure 4 shows the evaluation result of RDPNBC on the RDP database. (See Supp. for plot tables validating other methods on other databases; also See ATDmeasures.R and StatsandPlots.R in Supp. for implementation codes).

The top left plot shows that most sequences in the database resulted in correct predictions and around one-tenth of the sequences contained errors. Among the 12% error rate, 8% were singletons and 4% were non-singletons, which was compatible with the evaluation results from previous studies.

The top right plot shows the effect of switching from sequence count based metrics to taxon count based metrics. The overall error rate weighted by taxon count was 50%, showing that, though RDPNBC could correctly classify 88% of sequences in the database in cross-validation, those correct predictions only represent the capability of classifying half of the taxa. Here we see sequence count based metrics were biased toward the performance on majority taxa and failed to represent recognition capabilities.

The bottom left plot shows the effect of switching from binary error measurement to Taxonomy Distance. 88% of sequences had 0 TD corresponding to the 0-error sequences in the top left plot. Most of the 12% of sequences with errors were actually 0.16 TD (1-rank error). Here we see that Taxonomy Distance provides more detailed information on incorrect predictions and that singletons are not unavoidable errors.

The bottom right final ATD plot shows the ATDs across the taxa. We again see that most of the 0 TDs in the bottom left plot were from majority taxa and—though RDPNBC was perfectly correct on only half of the taxa in the database—most of the errors in the remaining taxa were 1-rank errors. The overall performance—ATD_by_taxa—was 0.11, showing expected half rank error for each prediction. The deployment of taxon count based metrics and Taxonomy Distance gave more robust and informative evaluation results.

### Method performance and best performance

There is a difference between “how good the method is” and “how close the method is to perfection”. By comparing the evaluation result to best performance, we can get the idea of how close a classifier is near to perfect and identify the difficult and important cases that algorithm designers need to work on. Here we describe how new metrics could work better for such a purpose.

When using binary error measurement, the behavior of the ideal (hereafter Plateau) algorithm can be described as: (1) If a taxon T is also presented in training data, predict T. (2) Else, get an error.

Considering Taxonomy Distance, the Plateau algorithm’s behavior can be defined in a more delicate form: (1) If a taxon T is also presented in training data, predict T. (2) Else, generate a prediction with min TD from taxonomy labels in training data.

Note that we use the verb “generate” to indicate that the prediction with the min TD was not necessarily the taxonomy label that had the min TD in training data. In some cases, the min TDs come from trimmed taxonomy labels. For example, suppose the training data contained only one single sequence with taxonomic label “orderA;familyB;genusC;speciesD”. When a classifier tries to make prediction on a sequence with the actual taxonomic label “orderA;familyB;genusE”, it can definitely not make an error-free prediction since there is no such taxonomic label in the training data. However, the best prediction with the smallest TD given the training data mentioned above was not “orderA;familyB;genusC;speciesD”, which would have 2/4 TD, but “trimmed” taxonomic label “orderA;familyB;” or “orderA;familyB;genusC” with 1/3 TD.

**Fig. 5:**
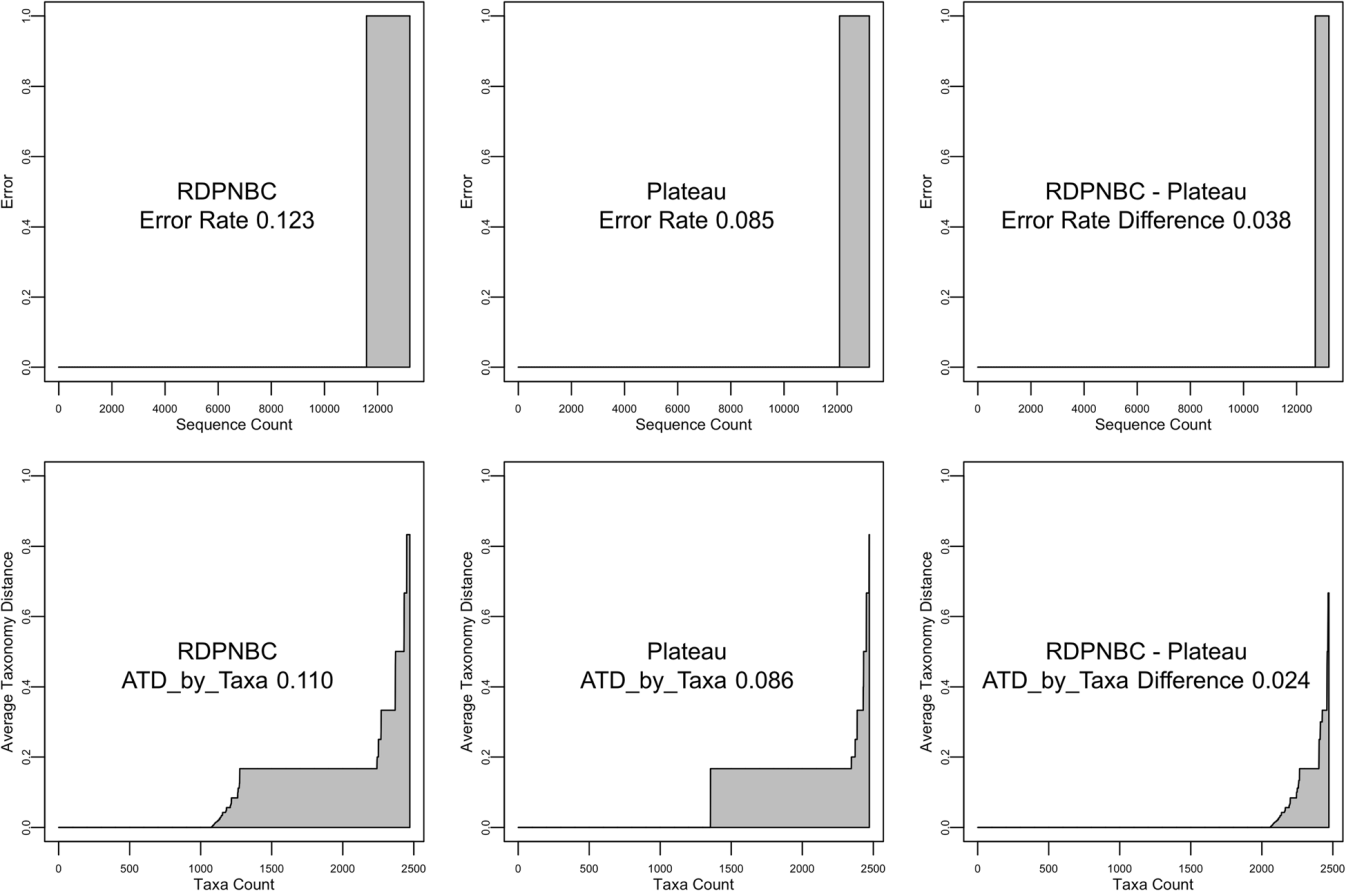
Evaluation results for the RDPNBC and Plateau methods using error rate and ATD

Figure 5 shows evaluation results for the RDPNBC and Plateau methods using error rate and ATD. From left to right, the first row shows the error for RDPNBC, Plateau and their difference. One could conclude “12% error for RDPNBC and 8% error for Plateau meaning 4% to improve.” The sequence count based metrics focused on having more correct prediction counts without the consideration for the overall recognition capabilities. Also, the deployment of binary error measurement does not provide incentive for taxonomic assignment methods to differentiate incorrect predictions.

The second row shows the ATD plots for RDPNBC, Plateau and their ATD differences (paired by each taxon). RDPNBC achieved 1/2 taxa error-free, 1/3 taxa one-rank error, and 4/5 of the taxa with error Plateau. For algorithm designers, this result not only points out what could or should be improved, but how much improvements may influence overall recognition capabilities. Here, we conclude ATD and ATD plot consider both recognition capabilities and correctness measure.

(See Supp. for testing other methods on other databases)

### Method performance comparison

**Fig. 6:**
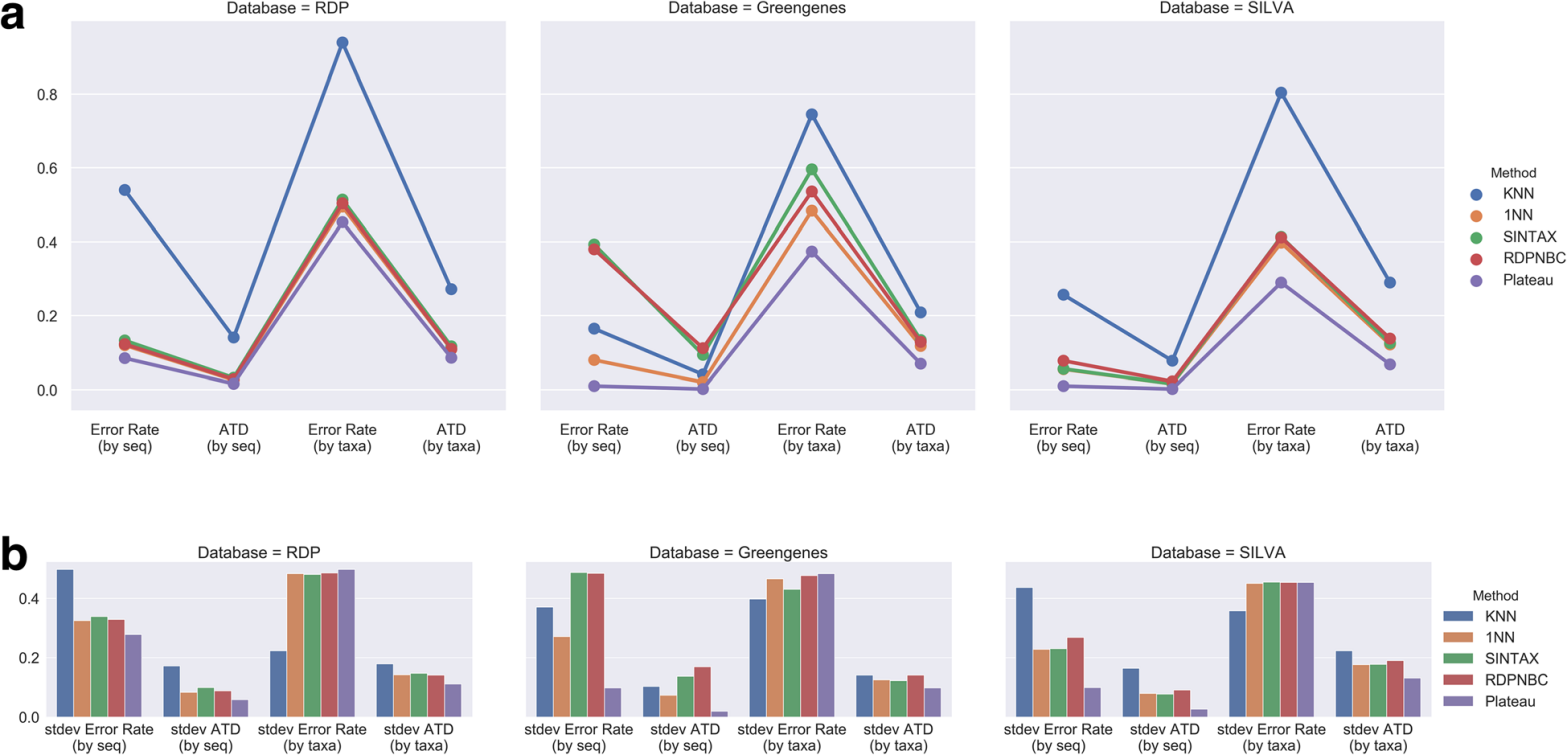
**a** Performances for four methods tested on three databases. **b** Standard deviation for each metric in (**a**)

Here we examine the effects of taxon count based metrics and Taxonomy Distance in method performance comparison. Error rate (by seq) is the simplest fraction of correctly predicted sequences; ATD (by seq) is a metric using Taxonomy Distance but without being taxon count based, making it simply the mean of TDs among the predictions. Error rate (by taxa) is a taxon count based metric that uses binary error measurement for each prediction, in which error rates are calculated for each taxon and then averaged. Figure 6 summarizes performance for testing the 4 methods on 3 databases.

The results of testing on RDP were compatible with experimental results in previous studies. All metrics show that the same performance ranking order and all methods, except KNN, were nearly equally good and closed to the Plateau.

When testing on Greengenes, Error rate (by seq) and ATD (by seq) showed that KNN significantly outperformed RDPNBC and SINTAX. However, standard deviations for these two metrics suggest that KNN is more prone to having unexpectedly large errors for some predictions than RDPNBC and SINTAX. Here, we see that binary error measurement leads to loss of information. On the other hand, KNN gets a decent 0.165 Error rate (by seq) but a high 0.745 error rate (by taxa). This shows that there is a high imbalance of taxa in Greengenes and sequence count based metrics favor methods that are good at recognizing majority taxa.

ATD_by_Taxa shows stable performance rankings “Plateau, 1NN, RDPNBC, SINTAX, KNN”, regardless of the databases used. There was still space for improvement.

**Fig. 7:**
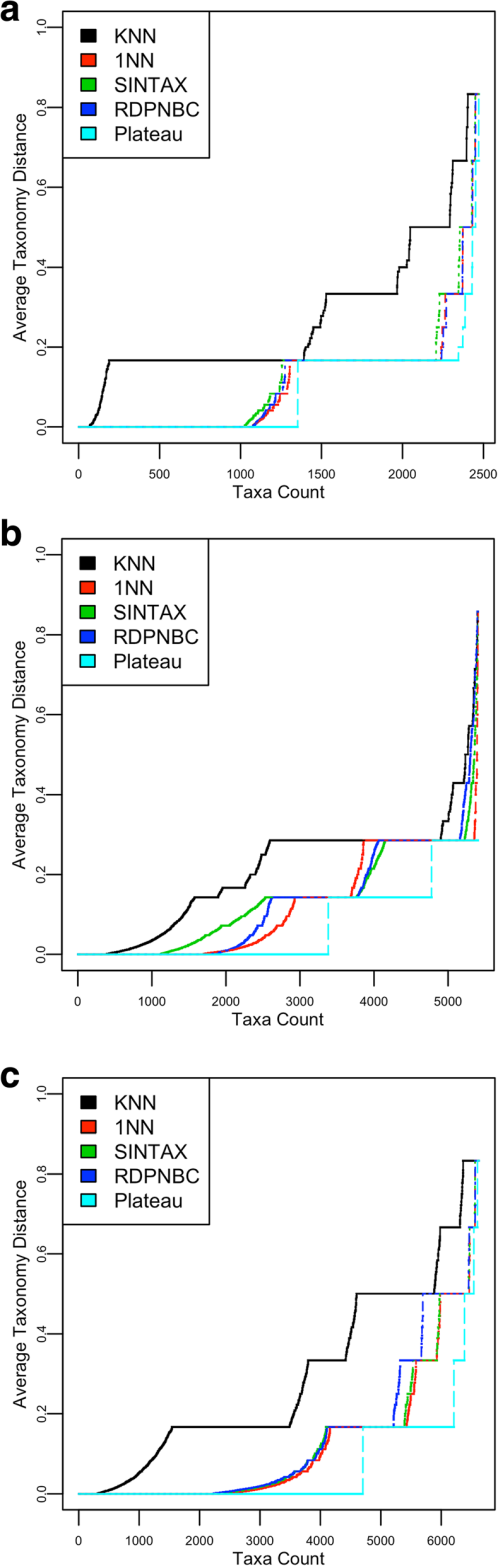
**a** ATD plots on RDP. **b** ATD plots on Greengenes. **c** ATD plots on SILVA

The merged ATD plots for the methods in Fig. 7 give more intuitive results for method comparison. To see which method performs better, one can simply ask “how close is the ATD line to the Plateau ATD line?” Fig. 7b and c show significant space for improving the tests on Greengenes and SILVA.

## Discussion

This study brings up taxa count based metrics and Taxonomy Distance to address the weaknesses in previous metrics. Kosmopoulos et al. [18] characterized existing metrics for evaluating HC algorithms into two classes: pair-based and set-based. Pair-based measures assign costs to pairs of predicted and true classes as the minimum distance in the tree hierarchy. Set-based measures are based on operations in the entire sets of predicted and true classes. The TD mainly uses the concept of set-based calculation.

The UniFrac metric shown in the taxonomic profiling challenge in Sczyrba et al.’s study [13] is more of taking the paired-based metric approach, calculating the minimum distance between the true taxonomic label and the predicted label in a taxonomic tree. Both Taxonomy Distance and UniFrac distance take advantage of the hierarchy in the taxonomic tree. Compared to UniFrac, TD puts more emphasis on higher-rank prediction errors, such as TD(T_4_, T_5_) > TD(T_1_, T_2_) in Table 2, and less on over-specialization cases. For example, suppose the actual taxonomic label is T_4_ in Table 2. T_6_ has 2 more lower ranks in the label than T_5_. The UniFrac distances for (T_4_, T_5_) and (T_4_, T_6_) are 2 and 4, respectively, being proportional to edge differences. On the other hand, TD(T_4_, T_5_) and TD(T_4_, T_6_) would be 1/2 and 3/4, respectively, reflecting more on rank differences.

Set-based HC metrics have hierarchical precision, recall and F-measure, as presented by Kosmopoulos et al. [18]. Nevertheless, hierarchical recall cannot reflect the over-specialization cases and hierarchical precision cannot reflect under-specialization ones. F-measure combines precision and recall but is less intuitive than TD, which centers around the concept of rank error.

However, TD also made the evaluation results highly dependent on taxonomy choice. The Taxonomy Distances might differ when using different databases. Some analysis platforms, such as Mothur, also made their own adjustments to taxonomic ranks. This also influences the calculation for Taxonomy Distance.

There are three things to notice when using Taxonomy Distance. First, we assume that the dissimilarity between taxa is proportional to their rank difference. Second, Taxonomy Distance is influenced by the number of ranks for the two taxa. Third, the concept of ATD is more like recall rate because the average is calculated by the true classes.

In addition to addressing concerns about taxonomy, for further studies we plan to evaluate performances of other taxonomic assignment methods, other biomarkers and other data sets. We also expect to make further biological interpretations based on those results.

## Conclusion

We conclude that the benefits of using taxon count based metrics and Taxonomy Distance in taxonomic assignment performance evaluation are as follow:

1. More robust: Taxon count based metrics give equal weight to each taxon and focus on recognition capabilities; they are therefore less prone to imbalanced databases.
2. More informative: Taxonomy Distance adopts the concept of taxonomic hierarchy and differentiates incorrect predictions.
3. More comparable: Taxon count based metrics solve the controversial problem of pruning large taxa and Taxonomy Distance clears the problem of whether to exclude singletons before or after testing.

The sequence count based metrics with binary error measurement used by previous studies imply the “same taxa abundance distribution to database” and “all different taxa are mutually equally different” assumptions. This makes performance evaluation and comparison results biased and less informative. This study proposes that ATD and ATD_by_Taxa, together with an ATD plot, avoid these problems.

## Data Availability

The data sets analyzed during the current study are available on Mothur’s wiki page. RDP: https://mothur.org/wiki/RDP_reference_files Greengenes: https://mothur.org/wiki/Greengenes-formatted_databases SILVA: https://mothur.org/wiki/Silva_reference_files There are 3. R files in Supp. used to run the experiment in this study: benchmarking.R: Cross-validated the user-chosen method on the database ATDmeasures.R: Calculated the measures for taxonomic assignment results StatsandPlots.R: Calculated the evaluation result files from ‘benchmarking.R ‘and plotted the figures in the paper mothur_to_usearch.sh: Convert taxonomy file(.tax) and template(.fasta) file from mothur.org into Usearch taxonomy format fasta file

## Abbreviations

1NN: Nearest Neighbor
ATD: Average Taxonomy Distance
HC: Hierarchical Classification
KNN: K-Nearest Neighbor
RDPNBC: RDP Naive Bayesian Classifier
TD: Taxonomy Distance
